# Is polymyxin B-immobilized fiber column ineffective for septic shock? A discussion on the press release for EUPHRATES trial

**DOI:** 10.1186/s40560-017-0236-x

**Published:** 2017-07-03

**Authors:** Toshiaki Iba, Lucy Fowler

**Affiliations:** 1 Juntendo University Graduate School of Medicine, 2-1-1 Hongo Bunkyo-ku, Tokyo, 113-8421 Japan; 2 University of Birmingham, Birmingham, UK

**Keywords:** Polymyxin B-immobilized fiber column, Sepsis, Septic shock, Randomized controlled trial, Endotoxin activity assay

## Abstract

The efficacy of polymyxin B-immobilized (PMX) fiber column on septic shock is still under debate. Recently, the result from “Evaluating the Use of Polymyxin B Hemoperfusion in a Randomized controlled trial of Adults Treated for Endotoxemia and Septic shock (EUPHRATES)” trial has been announced as a press release. According to that report, less than a 5% mortality difference was recognized in the “per protocol population” (*n* = 244, 31.9 vs. 36.9%) and the decrease was not statistically significant. However, among the patients in refractory shock with a multiple organ dysfunction score of more than 9 and an EAA between 0.6 and 0.9, a 10.7% reduction in 28-day mortality was recognized (*p* = 0.0474) when they received two sessions of hemoperfusion using the PMX fiber column. Since this favorable effect was obtained from “post hoc” analysis, further study is expected.

It is unfortunate that there has been no adjunctive therapy that has proven to be effective for sepsis and septic shock [1]. Japanese pharmaceutical companies and medical device makers have been intensively continuing the effort to develop a new drug or medical equipment to treat these life-threatening conditions [2, 3]. One of the most promising therapies is the use of hemoperfusion using a polymyxin B-immobilized (PMX) fiber column (Toraymyxin™). This device had previously been examined by two randomized controlled trials (RCTs); the first RCT, the EUPHAS trial [4], reported a favorable trend in terms of mortality in abdominal sepsis, while the second RCT named ABDO-MIX showed the opposite result [5]. Based on this evidence, “Japanese Clinical Practice Guidelines for Management of Sepsis and Septic Shock 2016” made a weak recommendation against the use of PMX for septic shock [http://www.jaam.jp/html/info/2016/pdf/J-SSCG2016_ver2.pdf#search=%27Japanese+Clinical+Practice+Guidelines+for+Management+of+Sepsis+and+Septic+Shock%27]. Following the aforementioned two RCTs, a third RCT, “Evaluating the Use of Polymyxin B Hemoperfusion in a Randomized controlled trial of Adults Treated for Endotoxemia and Septic shock (EUPHRATES),” has been conducted [6]. EUPHRATES was a multi-centered, placebo-controlled, and blinded trial performed in 50 ICUs in the USA and Canada. Patients with persistent septic shock despite adequate fluid resuscitation who were on vasopressors for more than 2 and less than 30 h were eligible for measurement with an endotoxin activity assay (EAA). Those with EAA ≥0.60 (intention to treat) were randomized to treatment with or without two sessions of PMX hemoperfusion (dummy columns were applied for the control patients). This trial started in October 2010 and intended to enroll 360 cases. However, the interim analysis suggested there was almost no effect in patients with a baseline multiple organ dysfunction (MOD) score of 9 or less, so these patients were excluded. As for the relationship between disease severity and efficacy of PMX, it is known that the effect becomes greater with increasing predicted mortality [7]. Indeed, the baseline mortality in EUPHAS (which showed PMX to be effective) was 53.3% and that in ABDO-MIX (which did not show PMX to be effective) was 19.5%. In EUPHRATES, the mortality in the control group was 36.4%. Thus, any negative result demonstrated in EUPHRATES could be because the disease severity was lower than initially expected. In the end, 450 cases were registered and the study was completed in July 2016.

A press release on 30 May has just announced the results of the EUPHRATES trial [http://www.spectraldx.com/assets/spectral-rls-05.30.17.pdf]. Initial results suggested a non-statistically significant reduction in 28-day mortality of less than 5% in the “per protocol population” (*n* = 244; MODS >9, EAA ≥0.6) when treated with two cycles of the PMX cartridge (*n* = 115) compared to control (*n* = 129). In post hoc analysis, a significant reduction in mortality was shown when the EAA is limited to less than 0.9, suggesting there is an upper limit to a patient’s pre-treatment burden of endotoxin in successful treatment with PMX. In 194 patients with MODS >9 and a baseline EAA between 0.6 and 0.9, there was a significant (*p* = 0.0474) 10.7% reduction in absolute 28-day mortality (26.1% in 88 patients in the PMX group vs. 36.8% in 106 patients in the control group), a relative reduction in mortality of 30%. The post hoc analysis also demonstrated that in patients where no bacteria could be identified on culture but who were highly endotoxemic (EAA 0.6–0.9), treatment with PMX was highly successful, showing a significant (*p* = 0.046) reduction in mortality from 42 to 21% compared to control. These patients have a higher baseline mortality, possibly due to a lack of microbiology targets meaning few other treatment options are available. However, the PMX treatment must be delivered before the results of blood culture are available, meaning in reality it will be impossible to tell which patients fall into this subgroup.

Though the information in the press release was limited, we think the EUPHRATES trial showed some positive results in the use of PMX in septic patients with endotoxemia. Interestingly, significant improvements in mean arterial pressure (*p* = 0.0462) and ventilation-free days (median difference of 14 days; *p* = 0.0043) were also reported between the groups. Since improvement in cardiovascular function is the fundamental mechanism by which this therapy is successful and the result was consistent with that of other reports [4], we think that hemoperfusion with PMX could achieve partial success. However, since the presence of both an MOD score of more than 9 and an EAA of less than 0.9 were not pre-fixed (original intention-to-treat) conditions, rational explanation should be requested to qualify this conclusion.

Another interesting point was the potential usefulness of EAA for patient selection. As mentioned before, the selection of the subjects is crucial for the success of the treatment. It has been reported that higher EAA was correlated with a higher risk of death in a clinical study [8]. Though the application of PMX is still under the debate, we think EAA can be useful for that purpose.

In summary, the EUPHRATES trial (per protocol analysis [refractory septic shock, EAA ≥0.60, two sessions of PMX and MOD score >9]) failed to show a positive result in the primary endpoint. However, there are still some positive points to take from the post hoc analysis, for example, improvement in survival and of cardiovascular function were observed. The results of the previous non-randomized studies were inconsistent. Some reported the favorable trends [9] and some reported the negative results [10]. The most recently published registry study [11] and meta-analysis has reported a favorable effect of this therapy [7, 12]. However, we think that such a high-cost therapy should have a clear prognostic effect before generalization.

In conclusion, EUPHRATES trial failed to show a positive result; however, some positive signals were recognized. Therefore, we think it is too early to give up the use of PMX. Since the effect was most evident in the subgroup of patients with abdominal infection with shock, an MOD score of more than 9 and an EAA of between 0.6 and 0.9, further study should be undertaken targeting this population.

## Abbreviations

EUPHRATES: Evaluating the Use of Polymyxin B Hemoperfusion in a Randomized controlled trial of Adults Treated for Endotoxemia and Septic shock
MOD: Multiple organ dysfunction
PMX: Polymyxin B-immobilized fiber column
RCT: Randomized controlled trial
